# Nrf2 Promotes Inflammation in Early Myocardial Ischemia-Reperfusion *via* Recruitment and Activation of Macrophages

**DOI:** 10.3389/fimmu.2021.763760

**Published:** 2021-11-30

**Authors:** Haijian Zhang, Yifei Liu, Xiaoqing Cao, Wenmiao Wang, Xiaohong Cui, Xuechao Yang, Yan Wang, Jiahai Shi

**Affiliations:** ^1^ Research Center of Clinical Medicine, Affiliated Hospital of Nantong University, Nantong, China; ^2^ Department of Thoracic Surgery, Nantong Key Laboratory of Translational Medicine in Cardiothoracic Diseases, and Research Institution of Translational Medicine in Cardiothoracic Diseases in Affiliated Hospital of Nantong University, Nantong, China; ^3^ Department of Pathology, Affiliated Hospital of Nantong University, Nantong, China; ^4^ Department of Thoracic Surgery, Beijing Chest Hospital, Capital Medical University (Beijing Tuberculosis and Thoracic Tumor Research Institute), Beijing, China; ^5^ Graduate School, Dalian Medical University, Dalian, China; ^6^ Department of General Surgery, Shanghai Electric Power Hospital, Shanghai, China; ^7^ Department of Emergency, Affiliated Hospital of Nantong University, Nantong, China

**Keywords:** early myocardial ischemia-reperfusion, Nrf2, Pdcd4, inflammation, macrophages

## Abstract

Cardiomyocyte apoptosis in response to inflammation is a primary cause of myocardial ischemia-reperfusion injury (IRI). Nuclear factor erythroid 2 like 2 (Nrf2) reportedly plays an important role in myocardial IRI, but the underlying mechanism remains obscure. Expression data from the normal heart tissues of mice or heart tissues treated with reperfusion for 6 h after ischemia (IR6h) were acquired from the GEO database; changes in biological function and infiltrating immune cells were analyzed. The binding between the molecules was verified by chromatin immunoprecipitation sequencing. Based on confirmation that early myocardial ischemia-reperfusion (myocardial ischemia/reperfusion for 6 hours, IR6h) promoted myocardial apoptosis and inflammatory response, we found that Nrf2, cooperating with Programmed Cell Death 4, promoted transcription initiation of C-C Motif Chemokine Ligand 3 (Ccl3) in myocardial tissues of mice treated with IR6h. Moreover, Ccl3 contributed to the high signature score of C-C motif chemokine receptor 1 (Ccr1)-positive macrophages. The high signature score of Ccr1-positive macrophages leads to the release of pro-inflammatory factors interleukin 1 beta and interleukin 6. This study is the first to elucidate the damaging effect of Nrf2 *via* remodeling of the immune microenvironment in early myocardial ischemia-reperfusion, which provides us with new perspectives and treatment strategies for myocardial ischemia-reperfusion.

## Introduction

Myocardial ischemia-reperfusion injury (MIRI) is defined as tissue damage that occurs when early and fast coronary flow returns to the heart after ischemia, which often exacerbates the damage caused by previous myocardial ischemia (1, 2). A growing number of studies have demonstrated that the reasons behind the condition mainly lie in three aspects: dysfunction of energy metabolism due to intracellular calcium overload, cardiomyocyte death due to increased oxygen free radicals, and cardiomyocyte death in response to inflammation (3). According to the time of reperfusion after ischemia, reperfusion can be divided into early (≤ 6 h) or late stages (4–7 days post-reperfusion) (4). During early reperfusion, a coordinated upregulation of inflammatory processes was observed in the ischemic area, whereas interstitial proteins, angiogenesis, and cardio-renal signaling processes were found to be increased at later reperfusion (days 4 and 7) (5). However, the mechanism of myocardial IRI, especially the role of the immune microenvironment in myocardial IRI, still needs to be further explored.

Nuclear Factor Erythroid 2 Like 2 (Nuclear factor erythroid 2-related factor 2; Nrf2; NFE2L2) is an important member of the cap´n´collar (CNC) basic leucine zipper family of transcription factors, characterized structurally by the presence of Nrf2-ECH (embedded contact homology) homology domains (6). Moreover, as the master regulator of the cellular oxidative stress response, Nrf2 regulates many cytoprotective genes and plays a central role in defense mechanisms against oxidative and electrophilic insults (7). Peroxisome proliferator-activated receptor-gamma co-activator 1α (PGC-1α), an important antioxidant molecule, interacts with Nrf2 to inhibit mitochondrial oxidative stress, promotes the clearance of damaged mitochondria, enhances mitochondrial biogenesis, and reduces the burden of IRI (8). Under oxidative stress, Kelch-like ECH-associated protein 1 (Keap1) is inactivated; Nrf2 escapes degradation based on the ubiquitin-proteasome pathway. Stabilized Nrf2 proteins form heterodimers with small Maf proteins in the nucleus and induce target gene expression for antioxidant reactions and detoxification by binding to antioxidant/electrophile response elements (9). Therefore, it plays an important role in the damage caused by oxidative stress. Please note, henceforth for the sake of uniformity, whether it’s human NRF2 or mouse Nrf2, it’s written Nrf2.

Programmed cell death 4 (Pdcd4), is a novel tumor suppressor to show multi-functions inhibiting cell growth, tumor invasion, metastasis, and inducing apoptosis (10). As a downstream target of miR-499a-5p, Pdcd4 reduced the infarct damage and cortical neuron apoptosis caused by I/R injury, which might represent a novel target that regulates brain injury by inhibiting Pdcd4-mediating apoptosis (11). In a rat model of IRI, overexpression of miR-21 in the heart could reduce cardiomyocyte apoptosis and myocardial infarct size, as well as protect the myocardium from ischemia/reperfusion by inhibiting Pdcd4 transcription (12). Additionally, a circular RNA, circPVT1, protects the myocardium from myocardial infarction (MI) and hypoxia/reoxygenation (H/R) injury by miR-125b/miR-200 sponges and then regulating Keap1/Nrf2- and Pdcd4-mediated apoptotic signaling (13). Pdcd4 contributes to c-Jun repression and p21 induction, and leads to the ubiquitination of Keap1-dependent Nrf2, which is the key factor of the cellular oxidative stress response (14). Hence, PDCD4 cooperates with Nrf2 to regulate apoptosis-related signaling pathways.

Myocardial ischemia-reperfusion (MI/R) promotes the polarization of proinflammatory M1 macrophages and aggravates heart injury (15). Macrophages promote endothelial-to-mesenchymal transition *via* MT1-MMP/TGFbeta1 after myocardial infarction (16). Macrophage AXL receptor tyrosine kinase worsens cardiac repair after reperfused myocardial infarction (17). Under the coating of platelet-like fusogenic liposomes (PLPs), mesoporous silica nanospheres with a payload of miR-21, an anti-inflammatory agent, can be specifically delivered to inflammatory monocytes in the blood circulation of MI/R-induced mice; they directly enter the cytoplasm of monocytes through membrane fusion, thereby realizing the reparative reprogramming of the inflamed macrophages derived from it. Ultimately, miRNA-21 delivered into macrophages *via* guidance and protection preserves the cardiac function of mice undergoing MI/R after being guided and protected (18). Hence, macrophages not only often play a pro-inflammatory role in myocardial IRI, but can also be reprogrammed to evolve as a therapeutic vector.

In this study, on the basis of confirming that early MI/R (MI/R for 6 h, IR6h) promoted myocardial apoptosis and inflammatory response, we hypothesize that Nrf2 promotes the transcription initiation of chemokines in the myocardial tissues of mice treated with IR6h, and then the latter contributes to the infiltration of pro-inflammatory M1 macrophages and the release of inflammatory factors. Therefore, we first investigated the inflammatory cells significantly associated with Nrf2 and Pdcd4 in the early stage of MI/R. Then, we explored the mechanisms by which Nrf2 promotes inflammatory cell infiltration. Finally, we investigated the effect of inflammatory cell infiltration on cardiomyocytes. We present the following article in accordance with the MDAR reporting checklist.^1^

## Materials and Methods

### Data Acquisition

Expression data from normal heart tissues of mice or heart tissues treated with reperfusion for 6 h after ischemia (IR6h) were acquired from the GEO database (No. GSE160516). The gene probe was transformed into gene symbols according to the annotation information for GPL23038 and normalized for differentiation analysis. Chromatin immunoprecipitation sequencing data of Nrf2 binding sites in human lymphoblastoid cells treated with anti- Nrf2 antibody or IgG antibody were downloaded from the GEO database (GSE37589); CHIP-seq data of A549 cells treated with Nrf2 siRNA or control siRNA with anti-H3K27ac antibodies were also downloaded from the GEO database (GSE118840).

### Analysis of Infiltrating Immune Cells

ImmuCellAI-mouse (immune cell abundance identifier for mice), a novel gene set signature-based method, is the mouse version of our previous ImmuCellAI (19). ImmuCellAI-mouse is a tool to estimate the abundance of 36 immune cells based on gene expression profiles from RNA-Seq or microarray data. It is worth noting that the 36 cell types were classified into three layers according to a hierarchical strategy.

### Gene Ontology Analysis and Kyoto Encyclopedia of Genes and Genomes Analysis

All differentially expressed genes, screened under conditions of P-value < 0.05, were subjected to GO analysis for allocation to relevant GO terms, including GO-BP (biological process), GO-CC (cellular component), and GO-MF (molecular function). Particularly, gene symbols of differentially expressed genes were converted to gene IDs according to the “org.Mm.eg.db” package; the latter were subsequently used for functional enrichment analysis according to the “clusterProfiler” package. Details of the genes belonging to particular GO terms with their fold change values are presented as circus plots using the “Goplot” package. As previous GO analysis, gene symbols of differentially expressed genes were converted to gene IDs according to the “org.Mm.eg.db” package; the latter were subsequently used for KEGG pathway analysis according to the “clusterProfiler” package.

### Gene Set Enrichment Analysis

The normalized expression data for all genes were acquired to conduct GSEA. GSEA was performed to identify the pathways that were significantly enriched between the IR6h and control groups according to the “clusterProfiler” package. If a gene set had a positive enrichment score, then the set was termed “enriched,” which implied that the majority of these genes in the set had higher expression and were accompanied by a higher risk score (20).

### Modeling of Myocardial Ischemia-Reperfusion, Immunohistochemical Evaluation

C57BL/6 mice aged 6-8 weeks were randomly divided into a sham operation group, ischemia group, and ischemia/reperfusion group, with 6 mice in each group. The mice were anesthetized by intraperitoneal injection of sodium pentobarbital (70 mg/kg) and placed on a heated mouse pad to prepare for surgery. The neck incision was opened, tracheal intubation performed under direct vision, it was confirmed that the intubation is in place, and a small ventilator was connected to assist breathing. Next, the mice in the ischemia group or ischemia/reperfusion group were opened to the left of the chest to expose the heart, and ligated the left anterior descending coronary artery (LAD) with silk thread 2mm below the left atrium to occlude it. After 30 min ischemia, the mice in the ischemia group were sacrificed and hearts were taken. Meanwhile, the mice in the ischemia/reperfusion group were terminated from ischemia by releasing the silk and the chest was closed. After 6 hours of reperfusion on a warming pad, the mice in the ischemia/reperfusion group were euthanized under anesthesia, and hearts were harvested. As for the sham operation group, the mice were euthanized and their hearts were harvested after endotracheal intubation and thoracotomy (21). Formalin-fixed paraffin-embedded heart tissues were cut at 2-3 μm and transferred to HistoGrip coated glass slides. The latter was performed out immunohistochemical staining by the labeled streptavidin-biotin method. The primary polyclonal Nrf2 antibody (Cat.AP52270, Abgent, USA; 1:500 dilution) was applied, 3,3’-Diaminobenzidine (DAB) was used as a chromogen for visualization of horseradish peroxidase activity, and hematoxylin was used as a counterstain. The NDP.View2 software was used to scan whole staining images of slides. The free software Image J (Java 1.8.0, 64 bit, https://imagej.nih.gov/ij) was used to independently analyze the percentage of Nrf2 positive staining on the whole slides by two pathologists (Yifei Liu and Wenmiao Wang).

### Transcriptome Sequencing

Rat cardiomyocyte H9C2 cells were divided into four groups with three replicates in each group. H9C2 cells in the hypoxia group were grown in low-glucose, serum-free medium and treated with hypoxia for 6 hours. After six hours of glucose deficiency and hypoxia, H9C2 cells in HR6h group or HR24h group were returned to normal culture in DMEM containing 10% fetal bovine serum (FBS) for 6 hours or 24h. As for control group, H9C2 cells in control group were harvested without any treatment. Subsequently, transcriptome sequencing (HiSeq X Ten) and analysis was performed at Vazyme Biotech Co., Ltd.

### Statistical Analysis

The genes included in the signature of C-C motif chemokine receptor 1 (Ccr1^+^) M1 macrophages are listed in **Table S1**. The signature scores, used to indicate the relative abundance of Ccr1^+^ M1 macrophages in each mouse myocardial sample, were calculated according to the formula: $\frac{\sum_{i=1}^{n}log_{2}\left(x_{ik}+1\right)}{n}$ , represents the number of signature genes listed in **Table S1**, k stands for sample included for analysis (22).

The differential expression between the two subgroups was analyzed by the “limma” package. The motif of the transcription factor is predicted by the “JASPAR2020” package. Chip-seq analysis was performed by the “GVIZ” package. The identification of up- or down-regulated genes was performed according to a set of screening criteria (P-value < 0.05). Continuous variables were compared using the t-test or Wilcoxon rank-sum test. Correlation analysis was performed using Pearson correlation. The grouping between the control group and IR6h group was broken down and reclassified into the high expression group and the low expression group according to the median value of specific gene expression or signature score, unless otherwise stated. All statistical analyses were performed using R software (version 4.0.4; R Foundation for Statistical Computing, Vienna, Austria). Statistical significance was set at P < 0.05; all significance tests were two-sided (23).

## Results

### Early Myocardial Ischemia-Reperfusion Promoted Myocardial Apoptosis and Inflammatory Response

**Figure 1:**
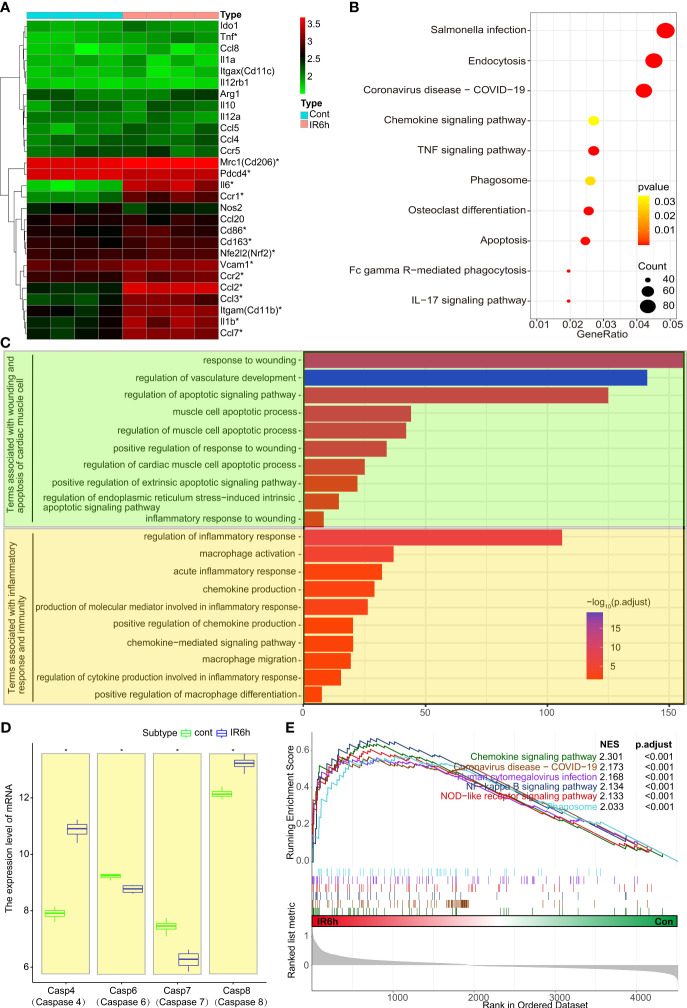
Early myocardial ischemia reperfusion promoted myocardial apoptosis and inflammatory response. **(A)** Heatmap of mouse inflammatory genes between the control group and IR6h group, including Nfe2l2 and Pdcd4. **(B)** KEGG - enriched signaling pathways according to differentially expressed genes. **(C)** GO-enriched modules of biological processes. **(D)** Transcriptional level analysis of caspase family members. **(E)** GSEA-enriched signaling pathway associated with inflammation or phagocytosis. IR6h, ischemia/reperfusion treatment for 6 h KEGG, Kyoto Encyclopedia of Genes and Genomes. GO, Gene Ontology. GSEA, Gene Set Enrichment Analysis. *P < 0.05, compared to the control group.

Our previous studies focused on the role of PDCD4 and Nrf2 in MI/R. We found that these two molecules play an important role primarily in inflammatory immunity (24). Therefore, we focused on the specific signature genes of macrophages and neutrophils. As shown in **Figure 1A**, the expression of mannose receptor C-type 1 (Mrc1, Cd206), C-C motif chemokine ligand 2 (Ccl2), C-C motif chemokine ligand 3 (Ccl3), C-C motif chemokine ligand 7 (Ccl7), integrin subunit alpha M (Itgam, Cd11b), interleukin 1 beta (Il1b), vascular cell adhesion molecule 1 (Vcam1), CD86 molecule (Cd86), CD163 molecule (Cd163), Nfe2l2 (Nrf2), Interleukin 6 (Il6), tumor necrosis factor (Tnf), and CCR1 was significantly upregulated in the IR6h group, compared with that in the control group. The expression of Pdcd4 was significantly downregulated (log_2_FC = -0.109, P = 0.028). The expression of Nrf2 in heart muscle tissue or cells under different conditions was shown in **Figure S1**. Whether the transcriptional analysis of mouse myocardial tissues (**Figure S1A**, GSE160516) or rat cardiomyocytes H9C2 (**Figure S1B**, OEP002796, we recently shared on a public data platform), the expression of Nfe2l2 (Nrf2) was significantly upregulated in the IR6h/HR6h group, compared with that in the control group. The immunohistochemical staining of Nrf2 Further confirmed the above conclusions (**Figure S1C** **)**. A thought-provoking phenomenon was that the expression of Nrf2 wasn’t always up-regulated or down-regulated during myocardial ischemia-reperfusion.

GSEA analysis (**Figure 1B**) showed that IR6h contributed to the enrichment of signaling pathways related to inflammation, such as “Salmonella infection, Coronavirus disease, chemokine/TNF/IL17 signaling pathways, and pathways related to apoptosis, including endocytosis, phagocytosis, osteoclast differentiation, and Fc gamma R-mediated phagocytosis. Consistent with the KEGG analysis, terms associated with wounding or apoptosis of cardiac muscle cells (prasinous), as well as terms associated with inflammatory response or macrophage-associated immune responses (orange) were enriched in the IR6h group, according to the GO analysis (**Figure 1C**). Noticeably, transcriptional level analysis of caspase family members suggested that the expressions of some apoptosis-related proteins including caspas8 were significantly increased in the IR6h group, compared with that in the control group (**Figure 1D**). Instead of the previous signaling pathway enrichment based on differential genes, enrichment of signaling pathways based on gene expression also suggested that IR6h resulted in the enrichment of signaling pathways related to inflammation (**Figure 1E**). Thus, these results suggest that MI/R for 6 h promotes myocardial apoptosis and inflammatory response.

### Nrf2 Cooperated With Pdcd4 to Promote the Infiltration of M1 Macrophages

**Figure 2:**
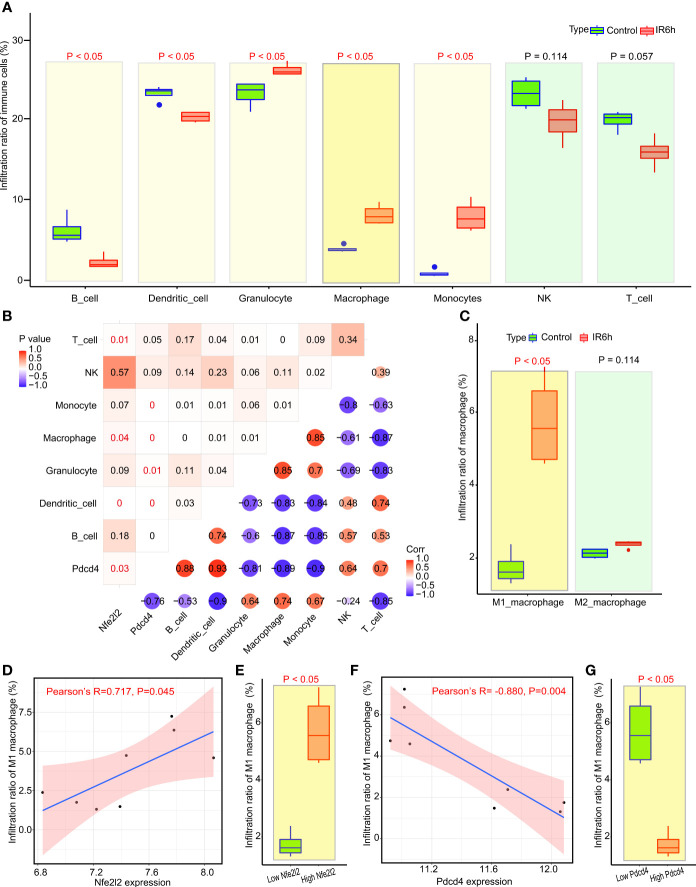
Nrf2 cooperated with Pdcd4 to promote infiltration of M1 macrophages. **(A)** Analysis of immune cell infiltration by ImmuCellAI. **(B)** Correlation analysis among seven categories of immune cells, Nfe2l2 and Pdcd4. The P-value is in the upper left corner; the corresponding Pearson correlation coefficient is in the lower right corner. **(C)** Subtype analysis of macrophages using ImmuCellAI. **(D)** Correlation analysis between M1 macrophages and Nfe2l2 expression. **(E)** Analysis of infiltrated M1 macrophages between the low Nfe2l2 and high Nfe2l2 groups. **(F)** Correlation analysis between M1 macrophages and Pdcd4 expression. **(G)** Analysis of infiltrated M1 macrophages between the low PDCD4 and high PDCD4 groups. ImmuCellAI, immune cell abundance identifier.

Since IR6h resulted in the enrichment of signaling pathways related to inflammatory and macrophage-associated immune responses, we analyzed the infiltration of immune cells in myocardial tissues. The proportion of immune cells infiltrating (%) according to ImmuCellAI-mouse was shown in **Table S2**. The first-level results of ImmuCellAI-mouse showed that the infiltration percentages of macrophages, granulocytes, and monocytes in myocardial tissues treated with IR6h were obviously upregulated, compared with those in the control group (**Figure 2A**). The percentage of immune cell infiltration in the second/third layers of ImmuCellAI-mouse analysis was shown in **Figure S2**. The proportion of all cells in the CD8^+^T or B subclasses was found to be decreased significantly in the IR6h group compared with that in the control group. Neutrophil was the only type of granulocyte significantly upregulated in the IR6h group compared with that in the control group (**Figure S2F**), although there was no significant difference in the proportion of overall granulocyte infiltration between the IR6h and control groups. However, there was no significant difference between Nrf2 expression and neutrophil infiltration, although there was a significant difference between Pdcd4 expression and neutrophil infiltration (**Figure S3**).

The correlation between Nrf2, Pdcd4, and immune cells is shown in **Figure 2B**. Macrophages are the only inflammatory immune cells associated with both Nrf2 and Pdcd4, and the expression of Nrf2 was positively correlated with the expression of PDCD4 (Pearson’s R = 0.7, P = 0.03). Subsequently, we observed (**Figure 2C**) that the infiltration rate of M1 macrophages, rather than that of M2 macrophages, was significantly upregulated in the IR6h group, compared with that in the control group. The grouping between the control group and IR6h group was broken down and reclassified into the high Nrf2 group and the low Nrf2 group according to the median value of Nrf2 expression. In fact, the expression of Nrf2 was positively correlated with the infiltration percentage of M1 macrophages in myocardial tissues; high expression of Nrf2 led to higher infiltration of M1 macrophages (**Figures 2D, E**). The grouping between the control group and IR6h group was broken down and reclassified into the high Pdcd4 group and the low Pdcd4 group according to the median value of Pdcd4 expression. In contrast, the expression of Pdcd4 was negatively correlated with the infiltration percentage of M1 macrophages in myocardial tissues; high expression of Pdcd4 led to lower infiltration of M1 macrophages (**Figures 2F, G**). In brief, Nrf2 promoted the infiltration of M1 macrophages, probably through a synergistic effect with Pdcd4.

### Nrf2 Cooperated With Pdcd4 to Promote the Transcription of Chemokines for Macrophages

**Figure 3:**
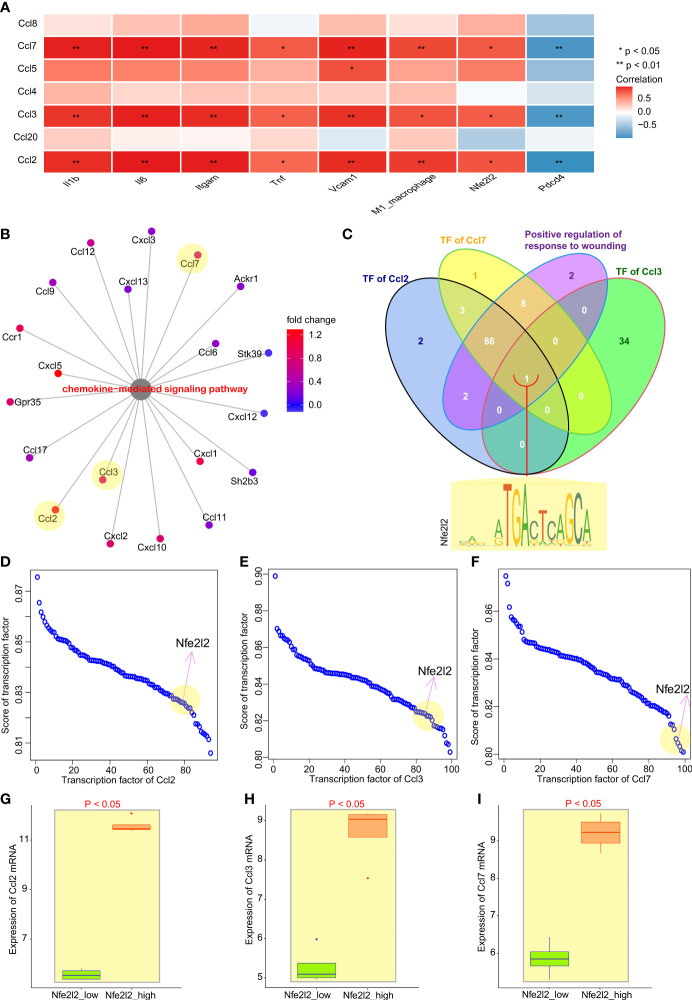
Nrf2 cooperated with Pdcd4 to promote transcription of chemokines for macrophages. **(A)** Correlation analysis between chemokines for macrophages and inflammation factors of M1 macrophages, as well as between Nfe2l2 and PDCD4. **(B)** Genes enriched in term “Chemokine-mediated signaling pathways”. **(C)** Venn diagram of transcription factors of Ccl2, Ccl3, or Ccl7, as well as genes enriched in term “Positive regulation of response to wounding”; Nfe2l2 was the only common gene. **(D–F)** Transcription factor score of Ccl2, Ccl3, or Ccl7. **(G–I)** Relationship between Nfe2l2 expression and the expression of Ccl2, Ccl3, and Ccl7.

To explore the mechanism by which Nrf2 promotes macrophage infiltration in myocardial tissues, we analyzed the correlation between chemokines for recruiting macrophages (25, 26) and inflammatory factors of M1 macrophages, as well as Nfe2l2 and Pdcd4. Correlation results showed (**Figure 3A**) that the expression of chemokines Ccl2, Ccl3, and Ccl7 was positively correlated with the infiltration rate of macrophages. Then, we analyzed the differential genes enriched in term of “Chemokine-mediated signaling pathways” resulting from IR6h; we found that chemokines Ccl2, Ccl3, and Ccl7 were all included in the term (**Figure 3B**). The venn diagram of transcription factors of Ccl2, Ccl3, and Ccl7, as well as genes enriched in term of “Positive regulation of response to wounding” (**Figure 3C**), suggested that Nrf2 (Nfe2l2) was the only transcription factor shared by chemokines Ccl2, Ccl3, and Ccl7. The transcription factors of these chemokines were sorted according to their scores in the JASPAR database (**Figures 3D–F**). The grouping between the control group and IR6h group was broken down and reclassified into the high Nrf2 group and the low Nrf2 group according to the median value of Nrf2 expression. Further analysis showed that Nrf2 promoted the expression of Ccl2, Ccl3, and Ccl7 (**Figures 3G–I**).

**Figure 4:**
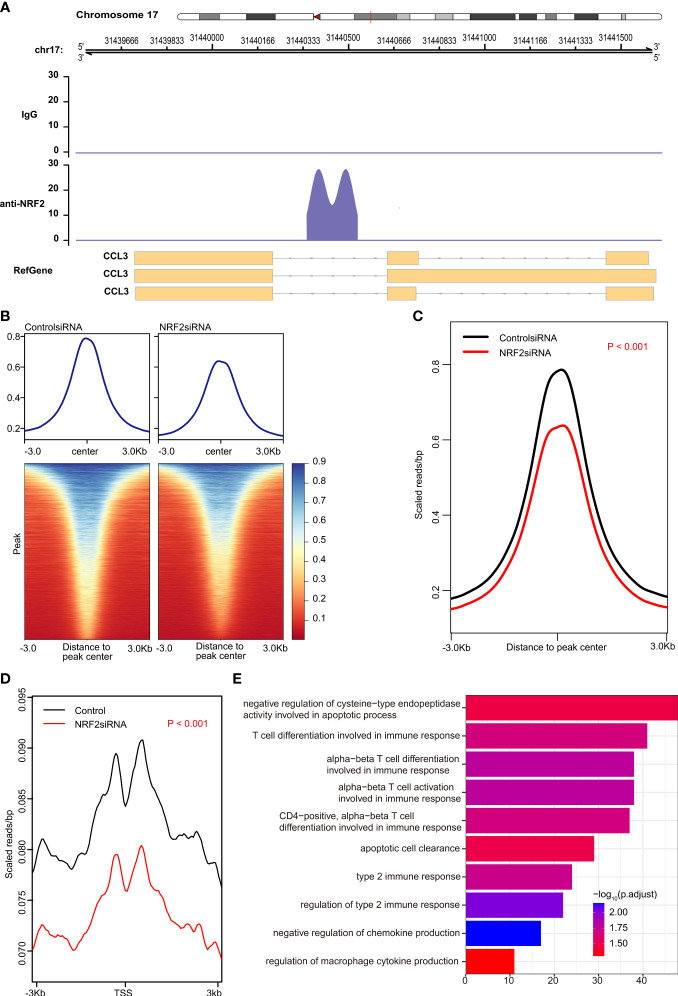
Nrf2 promoted transcription of Ccl3 validated by chromatin immunoprecipitation sequencing (CHIP-seq). **(A)** anti-NRF2 antibodies could bind to the DNA fragments of Ccl3, rather than to Ccl2 or Ccl7. **(B)** Heatmap of the peak signal. **(C)** The peak signal was significantly reduced in the NRF2 silencing group compared with the control group (P < 0.001). **(D)** The transcription start site (TSS) signal was significantly reduced in NRF2 silencing group, as compared with the control group (P < 0.001). **(E)** Gene Ontology (GO) analysis of peak id.

However, CHIP-seq is needed to determine whether Nrf2 binds directly to any chemokine. The results of CHIP-seq showed that anti-Nrf2 antibodies could bind to the DNA fragments of Ccl3, rather than to Ccl2 or Ccl7 (**Figure 4A**). In fact, both peak signal and transcription start site (TSS) signal were significantly reduced in the Nrf2 silencing group, as compared with those in the control group (P < 0.001) (**Figures 4B–D** **)**; Nrf2 silencing significantly promoted functional enrichment of T cell differentiation involved in immune response, apoptotic cell clearance, negative regulation of chemokine production, and regulation of macrophage cytokine production (**Figure 4E**).

### Nrf2 Promoted the Infiltration of M1 Macrophages and the Release of Inflammatory Factors *via* Initiating the Transcription of Ccl3

**Figure 5:**
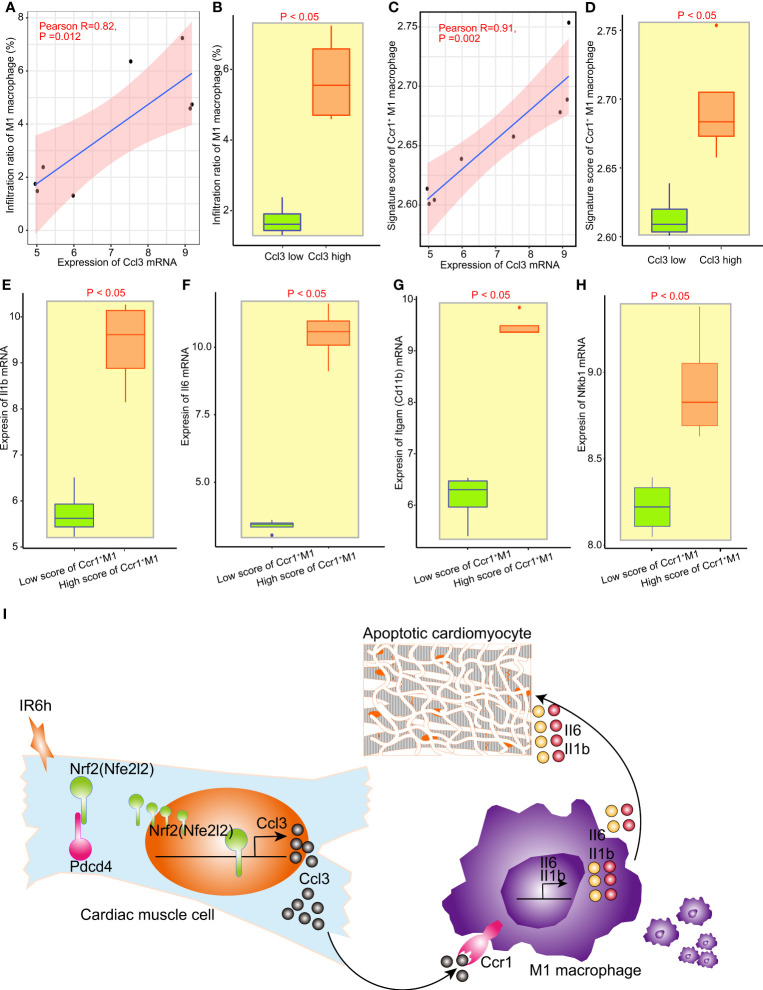
Nrf2 promoted the infiltration of M1 macrophages and release of inflammatory factors *via* initiating the transcription of Ccl3. **(A)** Correlation analysis between Ccl3 expression and M1 macrophage infiltration. **(B)** Analysis of infiltrated M1 macrophages between the low Ccl3 and high Ccl3 groups. **(C)** Correlation analysis between Ccl3 expression and the signature score of Ccr5^+^ M1 macrophages. **(D)** signature score of Ccr5^+^ M1 macrophages between the low Ccl3 and high Ccl3 groups. **(E–H)** The expression of inflammatory factors II1b, II16, Nfkb1, and macrophage labeling Cd11b between the low-score Ccr5^+^ M1 macrophage signature group and the high-score Ccr5^+^ M1 macrophage signature group. **(I)** Schematic diagram of Nrf2, cooperated with PDCD4, promoting the infiltration of M1 macrophages and release of inflammatory factors by initiating the transcription of Ccl3 in early myocardial ischemia reperfusion.

The grouping between the control group and IR6h group was broken down and reclassified into the high Ccl3 group and the low Ccl3 group according to the median value of Ccl3 expression. We found that the expression of Ccl3 was significantly positively correlated with the infiltration rate of M1 macrophages; high expression of Ccl3 led to higher infiltration of M1 macrophages (**Figures 5A, B**). Since Ccr1 is the only differentially expressed receptor for Ccl3, and Ccl3 was significantly positively correlated with M1 macrophages, we constructed a signature score of Ccr1^+^ M1 macrophages. As expected, the expression of Ccl3 was more strongly positively correlated with the signature score of Ccr1^+^ M1 macrophages, as compared to M1 macrophages (**Figures 5C, D**).

The grouping between the control group and IR6h group was broken down and reclassified into the high score of Ccr1^+^ M1 macrophages group and the low score of Ccr1^+^ M1 macrophages group according to the median value of signature score of Ccr1^+^ M1 macrophages. To assess the impact of Ccl3-recruited macrophages on myocardial tissue treated with IR6h, we evaluated the inflammatory factors released by Ccr1^+^ M1 macrophages. Results have suggested that the higher the score of Ccr1^+^ M1 macrophages, the higher the expression of Il1b, Il6, Itgam (Cd11b), and nuclear factor kappa B subunit 1 (Nfkb1) (**Figures 5E–H**). In other words, Ccl3-recruited macrophages resulted in the release of inflammatory factors such as Il-1b and Il-6.

## Discussion

In this study, on the basis of confirming that early MI/R (MI/R for 6 h, IR6h) promoted myocardial apoptosis and inflammatory response, we found that Nrf2, cooperating with Pdcd4, promoted the transcription initiation of Ccl3 in the myocardial tissues of mice treated with IR6h. Moreover, Ccl3 contributed to a high signature score of Ccr1-positive macrophages. The high signature score of the Ccr1-positive macrophages led to the release of pro-inflammatory factors Il-1β and Il-6 (**Figure 5I**).

In the early stages of MI/R, neutrophil-derived cathelicidin aggravates MI/R injury by over-activating TLR4 signaling and the P2X7R/NLRP3 inflammasome in heart-infiltrating neutrophils, which leads to the excessive secretion of IL-1β and subsequent inflammatory injury (27). N,N-dimethylsphingosine (DMS) has been documented to have protective effects against myocardial ischemia-reperfusion injury (IRI) by recruiting CD4(+)CD25(+)Foxp3(+) regulatory T cells (Tregs) *via* the PI3K/Akt pathway (28). The binding of CD137 on natural killer cells to CD137L on tubular epithelial cells triggers the production of the chemokine (C-X-C motif) receptor 2 ligands CXCL1 and CXCL2, and the subsequent induction of neutrophil recruitment, resulting in a cascade of proinflammatory events during kidney IRI (29). In this study, it’s Pdcd4 rather than Nrf2 that regulates neutrophil infiltration, but the specific regulatory mechanism needs to be further explored in the future. Of course, it cannot be ruled out that the lack of statistical difference in the correlation between Nrf2 and neutrophil infiltration may be due to the small number of samples, which need to be further explored in future studies (**Figures S2** **,** **S3**). Additionally, the following were observed in myocardial tissues treated with ischemia and reperfusion for 6 h: the expression of some macrophage-related genes was significantly upregulated (**Figure 1A**); the infiltration rate of macrophages was significantly increased (**Figure 2A**); the chemokines and phagocytosis-related signaling pathways were significantly activated (**Figures 1B, E**); and myocardial apoptosis-, inflammation-, and macrophage-related modules were abnormally activated (**Figures 1C, D**). Subsequently, our results suggested that, macrophages were the only inflammatory immune cells associated with both Nrf2 and PDCD4 (**Figures 2A, B**). Moreover, the infiltration rate of M1 macrophages, rather than that of M2 macrophages, was significantly upregulated in the IR6h group, as compared with the control group (**Figures 2C–G**). Eventually, we considered M1 macrophages as inflammatory immune cells associated with both Nrf2 and PDCD4, which was consistent with some previous studies. Fibrinogen-like protein 2 (FGL2) deficiency decreases macrophage infiltration and shifts the macrophage phenotype from a pro-inflammatory (M1) to an anti-inflammatory (M2) pattern in the early stage of coronary microvascular obstruction (29). Conversely, postponed activation of the M2a macrophage subpopulation resulted in a prolonged inflammatory response and adverse myocardial remodeling in CB2-deficient hearts (30).

Ccl2 secretion by epidermal keratinocytes is directly orchestrated by Nrf2, a prominent transcriptional regulator of tissue regeneration that is activated early after cutaneous injury. Epidermal keratinocyte-derived Ccl2 not only drives chemotaxis of macrophages into the wound, but also triggers macrophage expression of EGF, which in turn activates basal epidermal keratinocyte proliferation (31). Nrf2 knockout results in a decrease in Tnfα, Ccl3, and Cxcl2 gene transcription in zymosan-stimulated polymorphonuclear neutrophils, suggesting a role for Nrf2 in the regulation of proinflammatory cytokine production (32). Although our analysis indicated that Ccl2, Ccl3, and Ccl7 may all be potential genes directly transcribed by Nrf2 according to the authoritative JASPAR database (**Figure 3**), the analysis of available chromatin immunoprecipitation sequencing (ChIP-Seq) dependent on anti-Nrf2 antibody suggests that Ccl3, rather than Ccl2 or Ccl7, was found to directly initiate transcription by Nrf2 (**Figure 4A**).

Interestingly, Nrf2 knockout abolished the neuroprotective effects of SIRT6 overexpression (33). Nrf2 knockout suppressed the amelioration of cardiac function and histological alterations induced by myocardial ischemia-reperfusion injury after hyperbaric oxygen preconditioning, and increased oxidative products and proinflammatory cytokines *via* the PI3K/Akt pathway (34). Deficiency of Nrf2 suppressed cell proliferation, improved cell apoptosis with an increase in ROS and HO-1, and significantly increased the levels of chemokine 2 (Ccl2), interleukin-1β (IL-1β), tumor necrosis factor-alpha (TNF-α), angiotensin II receptor type 1 (AT1R), and reactive oxygen species (ROS) in the embryonic tissues, providing theoretical guidance for the application of Nrf2 in the treatment of preeclampsia (35). These findings are consistent with our analysis that Nrf2 silencing significantly promoted functional enrichment of T cell differentiation involved in immune response, apoptotic cell clearance, negative regulation of chemokine production, and regulation of macrophage cytokine production (**Figures 4B–D**).

A growing body of evidence suggests that Nrf2, as the upstream regulator of cytokine production, opposes transcriptional upregulation of proinflammatory cytokine genes and establishes a molecular basis for an Nrf2-mediated anti-inflammatory approach (36). Additionally, its protective role is associated with increased expression of antioxidant genes (ho-1, gclc, and nqo1) and decreased chemokine production (Ccl2, Ccl4, and Ccl11) (37). However, high Nrf2 was found to result in the upregulation of Ccl2, Ccl3, Ccl7 expression in the cardiac muscle tissues treated with IR6h (**Figures 3G–I**). These findings are completely contrary to those reports elaborating on its protective effects. The root of the contradiction with the existing understanding lies in that the perspective of the study may be different. Previous studies have focused on the cardiomyocyte level, while our current study mainly explores the relationship between multi-cells through deconvolution. Besides, a thought-provoking phenomenon was that the expression of Nrf2 was fluctuant, which was up-regulated during ischemia or early reperfusion and down-regulated during mid and late reperfusion.

Recruitment of monocytes into sites of inflammation is essential for immune response; Ccl2 is a major chemokine in the recruitment of monocyte-derived macrophages. Monocytes recruited *via* Ccl2/Ccr2, rather than Ccl5/Ccr5, propagate inflammation and tissue damage in osteoarthritis, thereby representing a promising therapeutic approach (38). However, Ccl3 also plays an important role in the recruitment of monocyte-derived macrophages. Ccl3 contributes to the formation of CD8^+^ T-cells and macrophage-enriched cardiac inflammation in chronic Trypanosoma cruzi infection; it creates a scenario with abundant IFNγ and TNF associated with cardiomyocyte injury, heart dysfunction, and QTc prolongation, which are biomarkers of Chagas heart disease (39). Pretreatment of wild-type mice with the specific Ccr1 antagonist BX471 also suppressed neutrophil and macrophage infiltration, suggesting a leukocyte source for these inflammatory chemokines and the existence of a Ccr1-dependent positive feedback loop for leukocyte infiltration in the model of renal ischemia-reperfusion injury (40). As expected, given that Nrf2 promoted Ccl3 expression rather than inhibiting it in the cardiac muscle tissues treated with IR6h, it is not surprising that Nrf2 subsequently promoted, rather than inhibited, macrophage infiltration (**Figures 5A, B**), especially the infiltration of Ccr1-positive macrophages (**Figures 5C, D**). Notably, Ccr5 is a receptor for Ccl3. However, since there was no significant difference in the expression of Ccr5 between the IR6h and control groups, we did not consider the infiltration of Ccr5^+^ macrophages, but directly considered the infiltration of Ccr1^+^ macrophages. In the tissue, immune cells trigger inflammation that, when uncontrolled, leads to fibrosis. Among the immune cells, macrophages play a special role in fibrosis formation, as macrophage depletion reflects less collagen deposition (41). Hence, a high score of Ccr1^+^ M1 macrophages resulted in a high expression of Il1b and Il6 (**Figures 5E–H**). In other words, Ccl3-recruited macrophages resulted in the release of inflammatory factors, which is consistent with our previous findings.

Admittedly, there are still some limitations to this study. Above all, we cannot completely deny the possibility that Nrf2 directly orchestrates the transcription of Ccl2 and Ccl7; the reasons are as follows: the samples for CHIP-seq analysis were not samples of mice treated with myocardial ischemia-reperfusion for 6h; CHIP-seq analysis dependent on anti-Nrf2 antibody does not necessarily reflect true and accurate results in the samples of mice treated with IR6h. Secondly, the conclusions of our study need to be further analyzed by flow cytometry, although the logic of our data analysis is quite rigorous. Thirdly, no article related to the data (No. GSE160516) has been published. The presented data did not provide the ischemia-reperfusion protocol, but according to conventional understanding, we can speculate that normal mice were treated with sham surgery, while mice in the IR6h group were subjected to ischemia for 30 mins with ligation of the left anterior descending coronary artery, subsequently subjected to reperfusion for 6 hours. As for the extent of myocardial injury, infarct size, and impaired contractile function, we may have to wait until the relevant data (No. GSE160516) is published to find a detailed explanation in their paper. However, although we only know 6 hours of reperfusion after ischemia and do not know more details, it does not affect the reliability of our results and conclusions, as well as the methods of data analysis. Fourthly, although caspase 8 was activated, caspase 3 and caspase 9 were not activated, the root cause of which may be that the pro-inflammatory macrophages recruited by Nrf2 did not promote myocardial apoptosis through mitochondria or death receptor pathways dependent on caspas3/9, but promoted apoptosis through apoptosis-inducing factor pathways. Fifthly, the root of the contradiction about the proinflammatory role of Nrf2 with the existing understanding lies in the fact that the expression of Nrf2 was fluctuant, which is up-regulated during ischemia or early reperfusion and down-regulated during mid and late reperfusion.

## Conclusion

In conclusion, our study found that Nrf2, cooperating with PDCD4, promoted the infiltration of M1 macrophages and release of proinflammatory cytokines Il1b and Il6 by initiating the transcription of Ccl3 in the myocardial tissues of mice treated with IR6h. This study is the first to elucidate the damaging effect of Nrf2 *via* remodeling of the immune microenvironment during early myocardial ischemia-reperfusion. Our findings may provide new perspectives and treatment strategies for myocardial ischemia-reperfusion. Our results may also be a powerful supplement to those reports previously elaborating the protective effect of Nrf2.

## Data Availability Statement

The datasets used during the current study and related scripts of bioinformatics analysis are available from the corresponding author on reasonable request. All data of OEP002796 can be viewed in NODE (http://www.biosino.org/node) by pasting the accession OEP002796 into the text search box or through the URL: http://www.biosino.org/node/project/detail/OEP002796.

## Ethics Statement

This study was reviewed and approved by the Animal Experiment Ethics Review Committee of Nantong University (S20210227-005).

## Author Contributions

Study concept and design: HZ and JS. Technical and material support: XQC, WW, XY, XHC, and YW. Analysis and interpretation of data, drafting of the manuscript: HZ and YL. Obtained funding and study supervision: HZ and JS. All authors contributed to the article and approved the submitted version.

## Funding

This work was supported by the National Natural Science Foundation of China (81770266); China Postdoctoral Science Foundation (2019M661907); Jiangsu Postdoctoral Science Foundation (2019K159, 2019Z153); General project of Jiangsu Provincial Health Committee (H2019101), Clinical Medical Research Center of Cardiothoracic Diseases in Nantong (HS2019001), Innovation Team of Cardiothoracic Disease in Affiliated Hospital of Nantong University (TECT-A04), and Nantong Key Laboratory of Translational Medicine of Cardiothoracic.

## Conflict of Interest

The authors declare that the research was conducted in the absence of any commercial or financial relationships that could be construed as a potential conflict of interest. 

## Publisher’s Note

All claims expressed in this article are solely those of the authors and do not necessarily represent those of their affiliated organizations, or those of the publisher, the editors and the reviewers. Any product that may be evaluated in this article, or claim that may be made by its manufacturer, is not guaranteed or endorsed by the publisher.

## Footnotes

1 Reporting Checklist: The authors have completed the MDAR reporting checklist.

## Supplementary Material

Supplementary Figure 1 The expression of Nrf2 in heart muscle tissue or cells under different conditions. **(A)** The expression of Nrf2 mRNA in mouse myocardium under different conditions according to the dataset of GSE160516. Control, Sham-operated group; IR6h/IR24h/IR72h, reperfusion for 6 hours/24 hours/72 hours after ischemia for 30 minutes. **(B)** The expression of Nrf2 mRNA in rat cardiomyocytes H9C2 under different conditions according to the dataset of OEP002796. Hypoxia, hypoxia for 30 minutes. Hypoxia, hypoxia for 30 minutes. HR6h/HR24h, reperfusion for 6 hours/24 hours after hypoxia for 30 minutes. **(C)** Typical image of anti-Nrf2 immunohistochemical staining in mouse myocardium under different conditions. Sham, Sham-operated group; Ischemia, ischemia for 30 minutes; IR6h, reperfusion for 6 hours after ischemia for 30 minutes. **(D)** Statistical analysis of immunohistochemical staining according to the positive rate of Nrf2.

Supplementary Figure 2 Percentage of immune cell infiltration in the second/third layers of ImmuCellAI-mouse analysis. **(A)** Schematic diagram of ImmuCellAI-mouse analysis. **(B–G)** Percentage of immune cell infiltration in T cell subclass, CD4^+^T cell subclass, CD8^+^T cell subclass, B cell subclass, CD4^+^T cell subclass, granulocytes subclass, or DC subclass.

Supplementary Figure 3 Correlation analysis between Neutrophil infiltration and the expression of Nfe2l2 or Pdcd4. **(A)** Correlation analysis between Neutrophil infiltration and the expression of Nfe2l2 (Nrf2). **(B)** Correlation analysis between Neutrophil infiltration and the expression of Pdcd4.

Supplementary Table 1 Gene list of Ccr1^+^ M1 macrophage signature.

Supplementary Table 2 The proportion of immune cells infiltrating (%) according to ImmuCellAI-mouse.
