# Supplementary figures and images for: Nrf2 Promotes Inflammation in Early Myocardial Ischemia-Reperfusion via Recruitment and Activation of Macrophages
Source: Front Immunol. 2021 Nov 30;12:763760. doi: 10.3389/fimmu.2021.763760 (PMC8669137; doi:10.3389/fimmu.2021.763760)

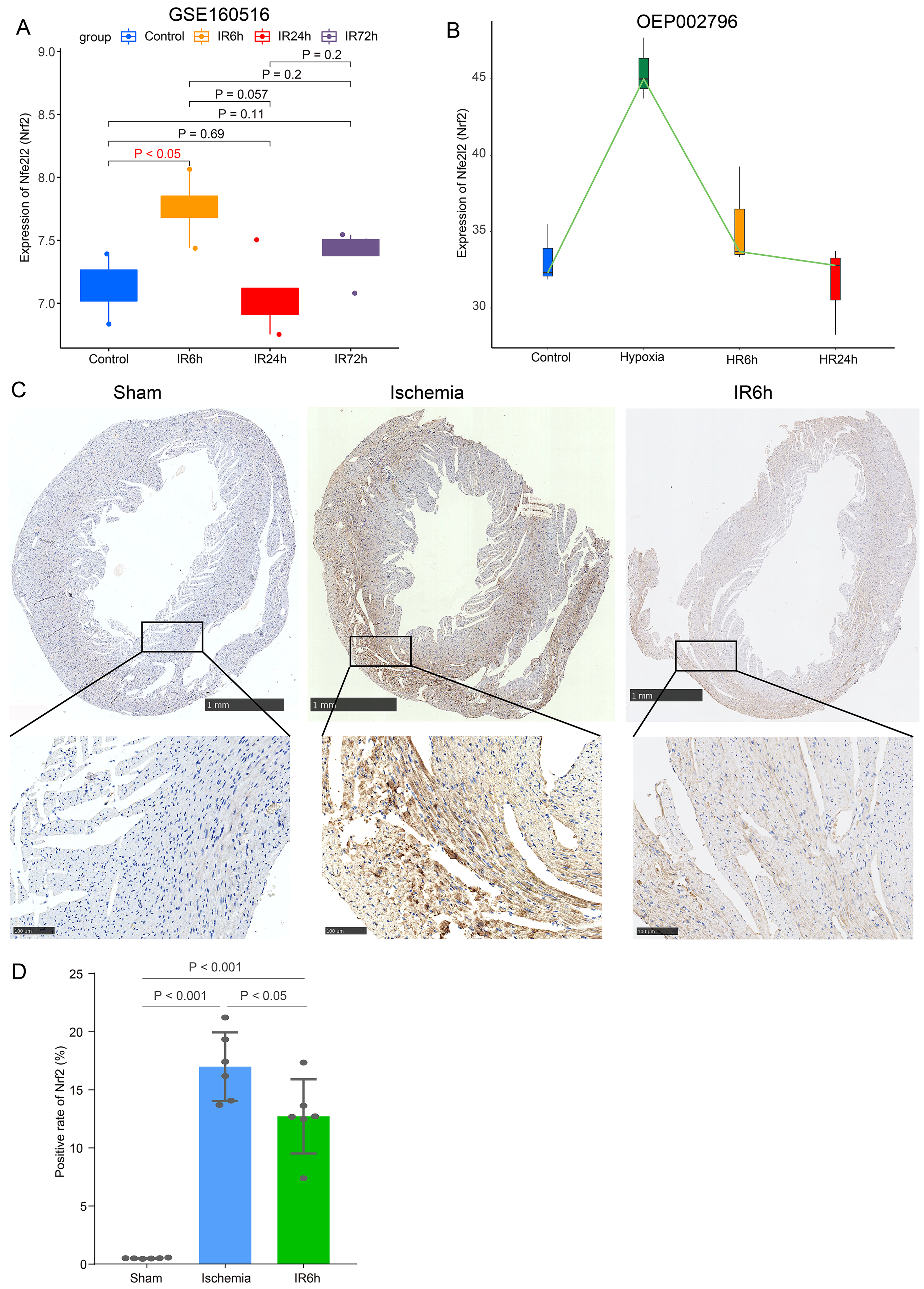

Supplement: Supplementary Figure 1 — The expression of Nrf2 in heart muscle tissue or cells under different conditions. (A) The expression of Nrf2 mRNA in mouse myocardium under different conditions according to the dataset of GSE160516. Control, Sham-operated group; IR6h/IR24h/IR72h, reperfusion for 6 hours/24 hours/72 hours after ischemia for 30 minutes. (B) The expression of Nrf2 mRNA in rat cardiomyocytes H9C2 under different conditions according to the dataset of OEP002796. Hypoxia, hypoxia for 30 minutes. Hypoxia, hypoxia for 30 minutes. HR6h/HR24h, reperfusion for 6 hours/24 hours after hypoxia for 30 minutes. (C) Typical image of anti-Nrf2 immunohistochemical staining in mouse myocardium under different conditions. Sham, Sham-operated group; Ischemia, ischemia for 30 minutes; IR6h, reperfusion for 6 hours after ischemia for 30 minutes. (D) Statistical analysis of immunohistochemical staining according to the positive rate of Nrf2. [file Image_1.tif]

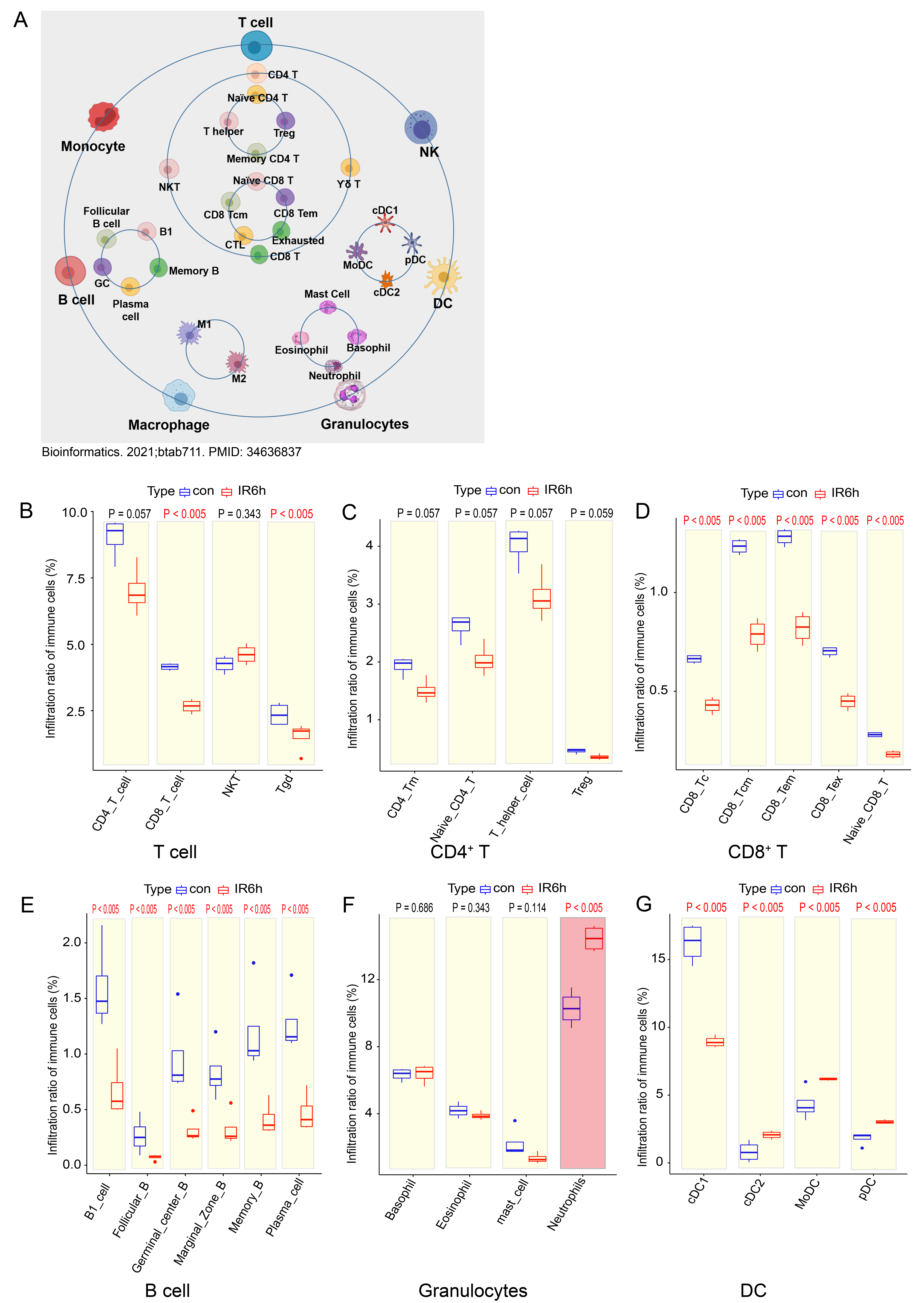

Supplement: Supplementary Figure 2 — Percentage of immune cell infiltration in the second/third layers of ImmuCellAI-mouse analysis. (A) Schematic diagram of ImmuCellAI-mouse analysis. (B–G) Percentage of immune cell infiltration in T cell subclass, CD4+T cell subclass, CD8+T cell subclass, B cell subclass, CD4+T cell subclass, granulocytes subclass, or DC subclass. [file Image_2.tif]
