# Supplementary material for: Nrf2 Promotes Inflammation in Early Myocardial Ischemia-Reperfusion via Recruitment and Activation of Macrophages
Source: Front Immunol. 2021 Nov 30;12:763760. doi: 10.3389/fimmu.2021.763760 (PMC8669137; doi:10.3389/fimmu.2021.763760)
Supplement: Supplementary Table 1 — Gene list of Ccr1+ M1 macrophage signature. [file Table_1.pdf]

Table S1. Gene list of Ccr1$^+$ M1 macrophage signature

Gene sets	Genes
**Ccr1$^+$ M1 macrophage**	Ache, Adamdec1, Apol6, Aqp9, Arrb1, Ccl19, Ccl5, Ccl8, Ccr7, Cd38, Cd40, Cxcl10, Cxcl11, Cxcl13, Cxcl9, Dhx58, Ebi3, Ggt5, Hesx1, Ido1, Kynu, Lag3, Lamp3, Nod2, Ptgir, Rassf4, Rsad2, Siglec1, Slamf1, Socs1, Tlr7, Tlr8, Tnfaip6, Tnip3, Trpm4, Ccr1
