# Supplementary material for: Nrf2 Promotes Inflammation in Early Myocardial Ischemia-Reperfusion via Recruitment and Activation of Macrophages
Source: Front Immunol. 2021 Nov 30;12:763760. doi: 10.3389/fimmu.2021.763760 (PMC8669137; doi:10.3389/fimmu.2021.763760)
Supplement: Supplementary Table 2 — The proportion of immune cells infiltrating (%) according to ImmuCellAI-mouse. [file Table_2.pdf]

Table S2. The proportion of immune cells infiltrating (%) according to ImmuCellAI-mouse.

id	GSM4874400	GSM4874401	GSM4874402	GSM4874403	GSM4874404	GSM4874405	GSM4874406	GSM4874407
B_cell_cells	5.93	4.79	5.2	8.73	1.69	3.54	1.72	2.13
Dendritic_cells_cells	21.92	23.41	23.78	23.49	20.77	19.78	20.8	19.54
Granulocytes_cells	22.86	24.27	20.86	24.3	25.53	25.53	27.16	26.04
Macrophage_cells	3.72	4.41	3.74	3.56	7.14	7.02	9.7	8.58
Monocytes_cells	0.68	0.54	1.46	0.7	10.31	6.15	6.57	8.63
NK_cells	25.12	21.74	24.49	21.23	16.38	22.3	20.7	19.02
T_cell_cells	19.77	20.83	20.48	18	18.18	15.68	13.35	16.06
CD4_T_cell_cells	9.04	9.52	9.58	7.92	8.28	6.97	6.08	6.73
CD8_T_cell_cells	4.28	4.08	4.24	4	2.93	2.54	2.36	2.82
NKT_cells	4.45	4.56	3.86	4.11	5.04	4.41	4.22	4.81
Tgd_cells	1.99	2.67	2.8	1.98	1.93	1.77	0.7	1.7
B1_cell_cells	1.55	1.27	1.4	2.16	0.5	1.05	0.51	0.64
Follicular_B_cells	0.48	0.09	0.2	0.3	0.03	0.09	0.08	0.08
Germinal_center_B_cells	0.86	0.74	0.76	1.54	0.26	0.49	0.24	0.27
Marginal_Zone_B_cells	0.79	0.59	0.76	1.2	0.25	0.56	0.22	0.27
Memory_B_cells	1.06	1	0.94	1.82	0.31	0.63	0.32	0.4
Plasma_cell_cells	1.18	1.1	1.13	1.71	0.34	0.72	0.35	0.47
cDC1_cells	17.35	15.48	14.53	17.51	9.47	8.68	9.08	8.54
cDC2_cells	0.33	1.7	1.2	0.05	2.22	1.92	2.37	1.72
MoDC_cells	3.15	4.16	5.99	3.97	6.15	6.25	6.27	6.07
pDC_cells	1.09	2.08	2.06	1.96	2.92	2.93	3.08	3.21
Basophil_cells	6.6	6.66	5.85	6.22	5.63	6.85	6.28	6.75
Eosinophil_cells	4.73	4.35	4.01	3.72	3.81	3.65	3.89	4.21

mast_cell_cells	1.77	1.73	1.89	3.59	1.07	1.34	1.78	1.2
Neutrophils_cells	9.77	11.53	9.11	10.77	15.02	13.7	15.2	13.88
M1_macrophage_cells	1.48	2.38	1.75	1.31	4.74	4.59	7.24	6.36
M2_macrophage_cells	2.24	2.03	1.99	2.25	2.4	2.43	2.46	2.22
CD4_Tm_cells	1.93	2.03	2.05	1.69	1.77	1.49	1.3	1.44
Naive_CD4_T_cells	2.62	2.76	2.77	2.29	2.4	2.02	1.76	1.95
T_helper_cell_cells	4.03	4.24	4.27	3.53	3.69	3.11	2.71	3
Treg_cells	0.46	0.49	0.49	0.4	0.42	0.36	0.31	0.34
CD8_Tc_cells	0.68	0.65	0.68	0.64	0.47	0.41	0.38	0.45
CD8_Tcm_cells	1.27	1.21	1.26	1.19	0.87	0.75	0.7	0.83
CD8_Tem_cells	1.32	1.26	1.31	1.23	0.9	0.78	0.73	0.87
CD8_Tex_cells	0.72	0.69	0.72	0.67	0.49	0.43	0.4	0.47
Naive_CD8_T_cells	0.29	0.27	0.29	0.27	0.2	0.17	0.16	0.19
Infiltration_score_cells	19.1	10	17.2	13	41.2	42.9	46.6	23.1
